# Vaginal Probiotics for Reproductive Health and Related Dysbiosis: Systematic Review and Meta-Analysis

**DOI:** 10.3390/jcm10071461

**Published:** 2021-04-02

**Authors:** Ana López-Moreno, Margarita Aguilera

**Affiliations:** 1 Department of Microbiology, Faculty of Pharmacy, Campus of Cartuja, University of Granada, 18071 Granada, Spain; 2 Instituto de Nutrición y Tecnología de los Alimentos, INYTA-Granada, 18100 Granada, Spain; 3 Instituto de Investigación Biosanitaria, Ibs-Granada, 18012 Granada, Spain

**Keywords:** vaginal probiotics, reproductive dysbiosis, bacterial vaginosis, VVC, IVF

## Abstract

The use of probiotics in reproductive-related dysbiosis is an area of continuous progress due to the growing interest from clinicians and patients suffering from recurrent reproductive microbiota disorders. An imbalance in the natural colonization sites related to reproductive health—vaginal, cervicovaginal, endometrial, and pregnancy-related altered microbiota—could play a decisive role in reproductive outcomes. Oral and vaginal administrations are in continuous discussion regarding the clinical effects pursued, but the oral route is used and studied more often despite the need for further transference to the colonization site. The aim of the present review was to retrieve the standardized protocols of vaginal probiotics commonly used for investigating their microbiota modulation capacities. Most of the studies selected focused on treating bacterial vaginosis (BV) as the most common dysbiosis; a few studies focused on vulvovaginal candidiasis (VVC) and on pretreatment during in vitro fertilization (IVF). Vaginal probiotic doses administered were similar to oral probiotics protocols, ranging from ≥10^7^ CFU/day to 2.5 × 10^10^ CFU/day, but were highly variable regarding the treatment duration timing. Moderate vaginal microbiota modulation was achieved; the relative abundance of abnormal microbiota decreased and *Lactobacillus* species increased.

## 1. Introduction

### 1.1. Microbiota Colonization Sites in Women’s Reproductive System

The taxa composition of the microbiota appears to exert a relevant role in reproductive and hormonal health, determining states of eubiosis versus dysbiosis [1]. The effects of microbiota imbalance seem to contribute to trigger reproductive [2,3], hormonal [4], and metabolic disorders [5,6]. Similarly, the reproductive-site microbiota can be affected by hormones or endocrine disruptor chemicals [7]. Conversely, if microbial dysbiosis occurs, subsequent decreased enzymes levels may diminish circulating estrogens and lead to recurrent reproductive pathologies [4,8]. Special attention has been paid to the following dysbiosis sites: vaginal, cervicovaginal, endometrial, and, indirectly, placental microbiotas. They are described in detail below.

The vaginal microbiota shows a specific colonization pattern for each woman. In the vaginal microbiota, the *Lactobacillus* genus is dominant and determinant during the establishment of a healthy microbiome community [9]. Recently, certain authors have postulated on the specific colonization of the endometrium. Specifically, a decrease in the *Lactobacillus* population appeared to be linked to implantation failures or early miscarriage in in vitro-fertilization patients [10,11]. However, there is controversy in these results and in determining the ratio of dominant microorganisms associated with health/dysbiosis. The theory of the existence of microbes in the placenta against the dogma of sterility has been experimentally approached by different authors. There is controversial research on the presence of specific microbiota in human uterine and placental sites and its effect on pregnancy and the fetus [12,13]. The hypothesis regarding the existence of microbiota in the placenta is generally considered disproven, as rigorously controlled studies found either pathogenic infections or no bacterial presence [12,13]. The formation and conservation of placental integrity and utility are known to be critical to fetal progress, and survival [14].

### 1.2. Microbial Dysbiosis Associated with Reproductive System Diseases

**Figure 1:**
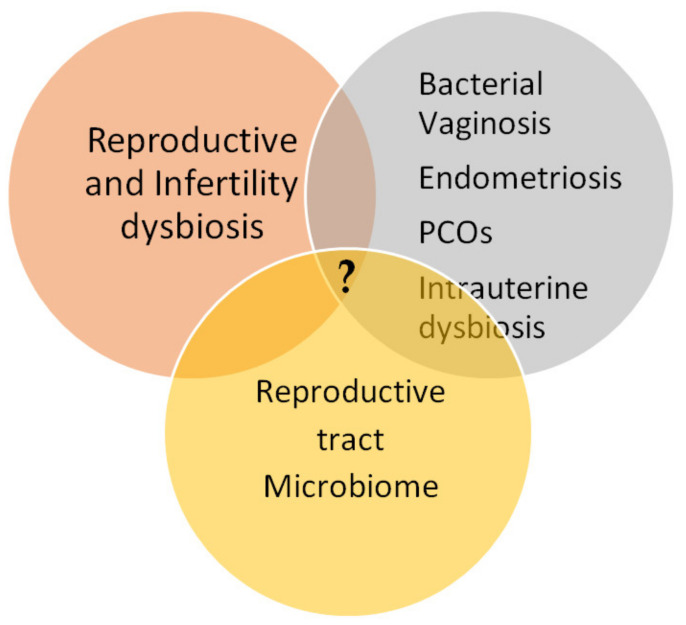
Intersection gap knowledge of reproductive system disorders, unexplained infertility, microbiome dysbiosis, and recurrent reproductive pathogenesis.

The reproductive tract microbiota’s composition and their variable patterns seem to be associated with alterations in reproductive disorders (Figure 1). Moreover, several recent studies have demonstrated that microbial dysbiosis could be linked to long-term recurrent reproductive modifications.

Bacterial vaginosis (BV) is the most prevalent reproductive disorder and is linked with gynecological complications, like spontaneous preterm labor, abortion, and endometriosis. It can be cured by restoring the representative vaginal components of the microbiota with probiotic formula, usually species of the genus *Lactobacillus* [15,16]. Salah et al. [17] postulated that BV is strongly implicated in underestimated causes of unexplained infertility. They found that BV detection and treatment improves the pregnancy rate in women [17]. Furthermore, van Oostrum et al. [18] claim that BV is meaningfully linked with preclinical pregnancy loss. They claimed that infertility is generally related to BV and atypical microbiota at lower genital tract, estimating that one in every five infertile patients suffers from BV and at least one in every three has an altered vaginal taxa microbiota composition. Thus, they suggested that BV might be involved in the etiology and irregular pregnancies of these patients.

In addition, among women of reproductive age, there are other common dysbiosis such as endometriosis, that have been linked to an unfavorable effect on fertility; 30% to 71% of women suffering infertility showed endometriosis and 30% to 50% of women with endometriosis are infertile [19]. Moreover, polycystic ovary syndrome (PCOS), linked to multiple physiological risk factors (obesity, hypertension, dyslipidemia, and insulin resistance), has also been associated with reproductive disorders [2,20]. Furthermore, spontaneous abortions and preterm deliveries, including non-implantation of the embryo, could be highly related to episodes of microbial dysbiosis; these could be modulated by restoring the disrupted microbiota [21,22,23,24]. Therefore, one strategy to counteract this bacterial misbalance involves the administration of probiotics, which are in indicative cases less harmful, safer and more natural than using antibiotics [22]. However, in general, studies of probiotics in relation to pregnancy complications generally require statistically significant sample sizes or complete data with a superior number of clinical trials and the determination of microbiota at the species and strains level [24].

### 1.3. Probiotics for Reproductive Health Interventions

The administration of probiotics for reproductive clinical translational studies are continuously progressing due to the growing interest in the previous scientific evidence reported for demonstrating the beneficial effects related to the restoration of natural microbiome colonization in reproductive sites.

Probiotics remain an important complementary intervention resource to modulate dysbiosis of the microbiota, which were associated with various metabolic disorders and diseases [25,26]. Therefore, specific doses of certain probiotic strains could modulate the microbiota toward a healthier state, that is, to recover the state of eubiosis [27,28]. Conversely, the inappropriate use of probiotics might pose some risks and safety concerns in immunologically compromised individuals [29].

### 1.4. Administration Routes of Probiotics in Reproductive Dysbiosis

Most clinical trials on the modulation of reproductive dysbiosis have been carried out using oral probiotics [30]. However, oral administration requires transfer of the probiotic bacteria to the site of colonization to promote a specific clinical effect, which implies that the probiotics have to subsist to the low pH of the upper gastrointestinal region. This transfer is generally demonstrated by the recovery of the specific microorganisms from fecal samples [25,31]. Specifically, in microbiota reproductive dysbiosis, probiotics should be transferred to the dysbiotic colonization sites, such as the vagina (vaginosis), the endometrium (endometritis), or the breast (mastitis). This transfer can be achieved via the physical ascending pathway, hematogenous route or lymph node transfer [32]. Presently, there are scientific results that prove benefits of probiotic microorganisms on reproductive health outcomes, such as the modulation of vaginosis [33], PCOS [20], and mastitis [34].

Vaginal administration of *Lactobacillus* can restore the vaginal microbiota by controlling the Nugent index within the range of normal values (0–3). Furthermore, *Lactobacillus* colonization is inversely correlated with the concentration of bacteria associated with bacterial vaginosis [35]. Moreover, to treat vulvovaginal infections, probiotics can be administered, preferably vaginally, to control the recolonization of *Lactobacillus* without any transfer needs or survival concerns towards the site of action [31].

The *Lactobacillus*-dominated endometrium may also benefit embryo implantation. However, there is controversy in these results and in determining the ratio of dominant microorganisms associated with health/dysbiosis status [36]. Furthermore, the same authors claimed that further taxonomical analysis of the endometrial microbiota may be necessary to identify and discern between the beneficial and/or pathogenic bacteria involved in embryo implantation. This would avoid multiple interventions against the anomalous microbiota that were not dominated by *Lactobacillus*.

Additionally, in a recent research, the link between endometrial microbiota composition and pregnancy outcomes in in vitro-fertilization (IVF) patients was examined. Remarkably, Moreno et al. [37] found an association between an endometrial microbial composition that was limited in *Lactobacillus* strains and ultimately adverse pregnancy outcomes. It was concluded that the negative effects of endometrial microbiota that are not dominated by *Lactobacillus* should be related with negative reproductive outcomes, such as implantation failure and pregnancy loss [38,39,40]. According to this, vaginal administration of probiotics could allow a direct, quicker, and targeted colonizing action to restore the altered vaginal microbiota compared to the long-term effects obtained by oral probiotics.

The main objective of the present work was to collect, scrutinize, and extract the most recent information from the high-quality and relevant scientific literature on probiotics administered vaginally and their possible qualitative and quantitative modulation capacities in reproductive-health-related dysbiosis.

## 2. Materials and Methods

### 2.1. Eligibility Criteria and Search Strategy

All interventional studies compiling data on specific probiotic microbial strains and dosages administered for human reproductive microbiota-related dysbiosis were included. Two reviewers, ALM and MA, screened titles, abstracts, and then full-text papers independently against inclusion criteria according to Preferred Reporting Items for Systematic Reviews and Meta-Analyses (PRISMA) [41].

These four criteria were applied for the selection of the study: (1) being published within the last fifteen years, specifying (2) the probiotic strain used, (3) the dose, and (4) the time/period of administration. The specific data on population, intervention, comparison, and outcome criteria for inclusion in the comprehensive review are described in Table 1.

Non-English-language manuscripts and documents or studies without specific data on fertility and reproductive dysbiosis biomarkers were excluded.

Each eligible article identified was reanalyzed by its title and abstract, and the eligible articles were selected for complete reading. The initial selection was done based on a designed term search through title and abstract screening, and the second selection was based on a full-text screening, where the two independent reviewers revised the publications with specific reference to the inclusion criteria. The study selection interrater agreement between the two reviewers was calculated as the proportion of positive agreement (PA) [42].

**Figure 2:**
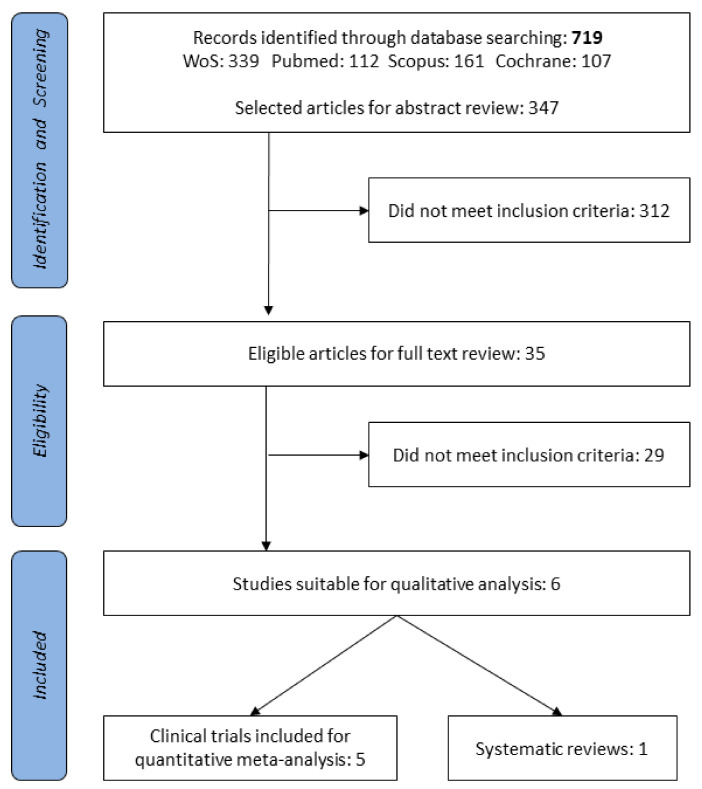
Vaginal probiotics Preferred Reporting Items for Systematic Reviews and Meta-Analyses (PRISMA). Complementary oral probiotics PRISMA was previously performed [45].

Literature search and review were carried out under the stepwise search procedure. The systematic review was developed in collaboration with University of Granada library support using search keywords/terms (described below) and medical subject headings (MeSH). MEDLINE/PubMed [43], Web of Science (Thomson Reuters Scientific, Philadelphia, PA, USA), Scopus (Elsevier, Amsterdam, The Netherlands), and Cochrane Library [44] were the databases used. A PRISMA flow diagram of the literature search condenses the selection of the studies comprising the two screening phases (Figure 2).

The collective search approach was carried out using MeSH and free text search terms detailed as follows: (probiotic* and infertility and doses); (probiotic* and microbiota and fertility); (probiotic* and microbiota and infertility); (probiotic* and “vaginal microbiota” and infertility); (probiotic* and endometriosis); (probiotic* and endometriosis and fertility); (probiotic* and endometriosis and infertility); (probiotic* and “endometrial microbiota” and infertility); (probiotic* and endometrium and infertility); (probiotic* and endometrium and fertility); (probiotic* and microbiota and “*vaginal administration”); (probiotic* and ovules); (probiotic* and reproductive and “*vaginal administration”); (probiotic* and “Polycystic Ovary Syndrome”).

### 2.2. Data Extraction, Analysis, and Risk of Bias (Quality) Assessment

The resulting data were extracted from all the selected clinical studies: publication year, study design, characteristics of the population and sample size (n) in the intervention group, sex, and age; microorganism probiotic strains; doses and pattern of administration; modification of the main clinical outcomes, Nugent score, or alterations in several fertility-related parameters. The main data results extracted from CT were qualitatively compiled and organized into form of table detailed below in the results.

**Figure 3:**
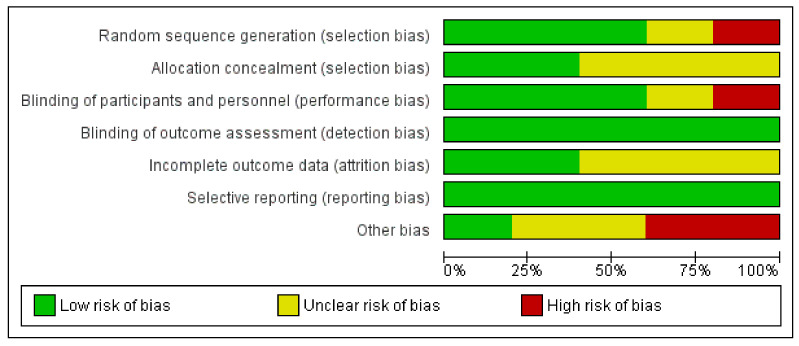
Risk of bias graph of clinical trial (CT): review authors’ judgments about each item as percentages across all included studies.

**Figure 4:**
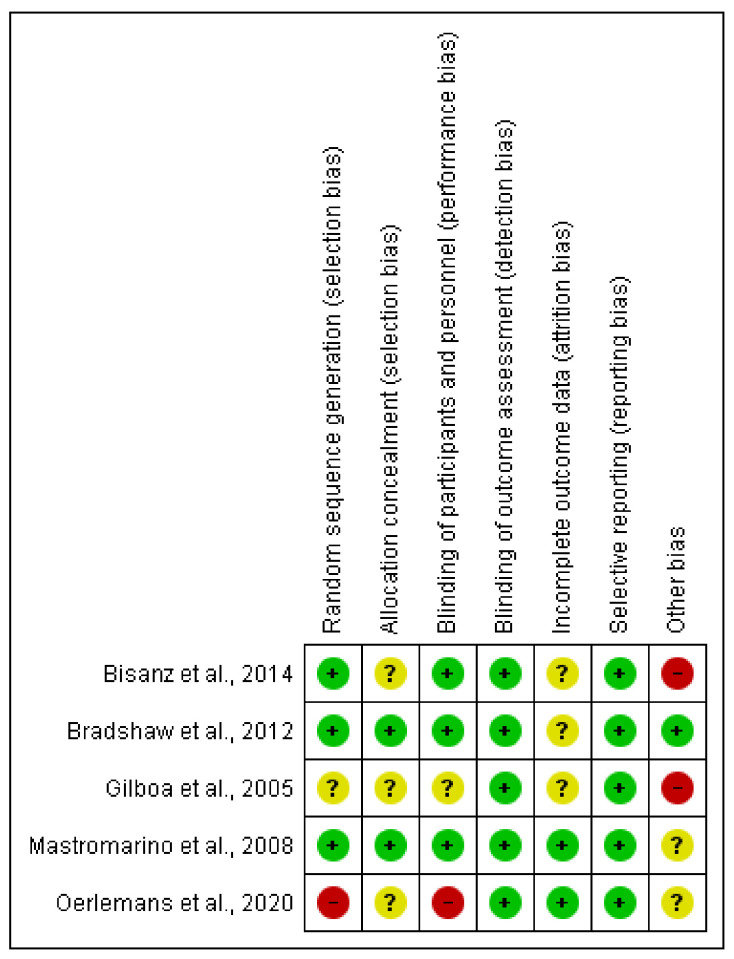
Risk of bias summary of CT: authors’ judgments about each risk of bias item for each included study low risk (+, green circle), high risk (−, red circle), or unclear risk (?, yellow circle).

The risk of bias for each clinical trial selected was assessed independently by the authors using the Cochrane collaboration methodology [44]. The risk of bias was tabulated for each study (Figure 3 and Figure 4). Each item evaluated was classified as low risk, high risk, or unclear risk according to the quality recommendations described in Chapter 8 of the Cochrane Handbook of Systematic Reviews of Interventions [44]. Analysis and corresponding figures were generated in RevMan 5.3 Review Manager (RevMan Computer program) Version 5.3. Copenhagen: The Nordic Cochrane Centre, the Cochrane Collaboration, 2019, available at (revman.cochrane.org accessed on 20 January 2021).

### 2.3. Statistical Analysis

To compute the global quantitative effect for each relevant study analyzed regarding the modulation of vaginal microbiota capacities, the subsequent phases were carried out: (1) Extraction data regarding the baseline value in treatment group, baseline value in placebo group, endpoint in treatment group, and endpoint value in placebo group. When baseline values were not stated, only the endpoints were used. (2) Value change ± SD from baseline was calculated for the treatment and placebo groups, separately. (3) The mean variance between data from baseline in probiotics group versus placebo group was calculated and used as the overall effect size.

Alignment calculations and Hedges’ adjusted *g* were used to calculate the effect size. A random-effects model pooled the calculated effect sizes. Heterogeneity was explored using the I^2^ test and considering I^2^ > 75% high heterogeneity and I^2^ < 25% low heterogeneity. Heterogeneity between subgroups was calculated using a fixed-effects model. Sensitivity analysis was executed by omitting one study at a time to detect any significant changes in the results obtained. Begg’s rank correlation test and Egger’s regression asymmetry test were used to assess publication bias.

## 3. Results

Reproductive disorders are an increasing global health concern. Therefore, the plausible role of the microbiota in reproductive and hormonal health has promoted studies administering vaginal probiotics. Accordingly, an initial search with the keywords “probiotics and fertility” showed a triplication of available studies over the last fifteen years. A total of 719 documents were retrieved as a result of applying the selection criteria. A total of 35 clinical studies eligible for vaginal probiotics were selected for full-text review (Figure 2). The positive agreement (PA) value was 0.85 for titles and 0.90 for abstracts. When the full texts were analyzed for the specific strain, doses, and patterns, only six articles (clinical trials (*n* = 5) and a systematic review (*n* = 1)) fulfilled the inclusion criteria. The carefully chosen and included studies were extensively analyzed and the relevant qualitative outcomes are shown in Table 2.

The qualitative comparative data were extracted based on the following categories: sample number, population characteristics, probiotic strain(s), dosage and administration patterns, intervention period (weeks), disorder treated, and modulation data of clinical outcomes related with fertility disorders (Table 2).

The quality of the selected clinical studies was guaranteed based on the comprehensive method applied in the selection of the final documents and their outcomes in order to obtain comparative and useful conclusions. To evaluate the five clinical trials (CTs) in terms of their design, execution, and outcomes, the risk of bias was evaluated (Figure 3 and Figure 4), increasing the classification of the quality standards and giving extra significance to the selected CTs, as well as allowing the validation of the revised results. The disorders treated in the selected CTs were one of vulvovaginal candidiasis, two of BV, one of recurrent BV, and one of IVF (Table 2). The selected systematic review [46] only contained one article [47] that met the established inclusion criteria; however, the available data did not contain enough specifications to be incorporated into the quantitative analyses. The qualitative information extracted was aligned with normal microbiota restoration effects. The administration of *L. acidophilus* KS400 (Gynoflor^®^) to 360 women with BV did not show a significant improvement in BV symptoms, although the normal flora index (NFI) augmented meaningfully in the treated group. Further limitations beyond the restrictive design of the systematic review include the lack of studies fulfilling the strict inclusion criteria.

**Figure 5:**
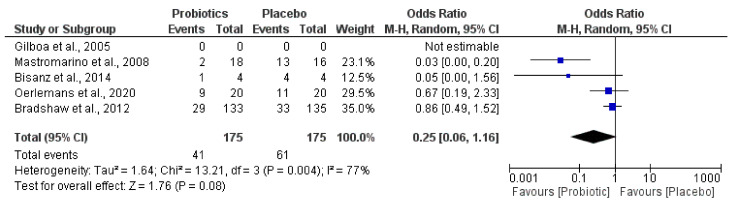
Effect of vaginal probiotics for modulation reduction in abnormal microbiota.

**Figure 6:**
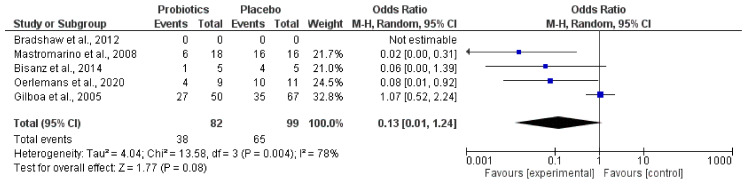
Effect of vaginal probiotics for modulation increase in *Lactobacillus* spp.

Furthermore, the most relevant changes and modulation capacities of vaginal probiotics administered on the abnormal microbiota (Figure 5) and the *Lactobacillus* genus amount (Figure 6) were revealed by quantitative examination through forest plot assessments, where the statistical impact on clinically significant parameters was verified.

The quantitative outcome promoted by the diverse probiotics administered in each population studied in relation to the capacity for a reduction in abnormal microbiota and the increase in the *Lactobacillus* genus amount is indicated by black diamonds.

Interestingly, the meta-analysis showed that probiotics groups could reduce the amount of abnormal microbiota (*Gardnerella* and *Atopobium*) (Figure 5) and increase in parallel the quantity of species belonging to the *Lactobacillus* genus (Figure 6).

## 4. Discussion

In the last several decades, reproductive disorders and infertility cases have increased. This seems to be the result of multiple factors and hormonal imbalances triggered by different etiologies including polycystic ovary syndrome (PCOS), endometriosis, obesity or metabolic syndrome, bacterial vaginosis, infections, and even some cancers [2]. Recently, most of these metabolic disorders have been concomitantly linked to reproductive microbiota dysbiosis [2]. Consequently, many studies were conducted to establishing the healthy female reproductive microbiota, and its role in fertility dysbiosis [8,9,10]. Healthy microbiota at reproductive sites contains lactobacilli as the most represented bacteria, but other anaerobic genera might be present such as the genera *Prevotella*, *Gardnerella*, *Atopobium*, *Megasphaera*, *Sneathia*, and *Anaerococcus* [53,54,55,56]. All these bacteria seem to also be involved in diverse phases of reproduction such as gamete formation, fertilization, gestation establishment, and maintenance, and also in the bacterial transfer mother-newborn [56,57]. There are multiple factors that act modifying the reproductive tract microbiome equilibrium, mainly triggering bacterial vaginosis as the most reported dysbiosis [58]. However, we highlight the misuse of antibiotics, together with cumulative exposure to several xenobiotics, and endocrine disruptors, which can also influence the healthy microbiome [7], especially when exposure occurred via direct contact with a high level of contaminants in hygiene products [59,60].

Our study highlights the probiotic modulation capacities in relation to bacterial vaginosis, which showed the restoration of a healthy microbiome. *Lactobacillus* spp. were the dominant colonizers in reproductive sites [38] and defended these sites against abnormal or pathogenic microorganisms [61]. Accordingly, more probiotic interventional studies have been conducted on reproductive failures with lactobacilli imbalance, such as adverse pregnancy outcomes [16], a significant decrease in endometrial implantation [36], and altered IVF outcomes [62,63]. The *Lactobacillus* genus has optimal probiotic properties, including high hydrophobicity and self-regulation, adhesion to epithelial cells and acid production [64], and restoration of healthy urogenital microbiota [65,66,67]. In this systematic review, combinations of *Lactobacillus* strains were also administered in most studies, as *L. acidophilus* KS400 was present in all formulae administered as single probiotic strain. Importantly, this strain produced bacteriocins with antimicrobial activity against relevant urogenital pathogens [68]. In addition, a combination of strains were administered orally and vaginally, similar to *L. rhamnosus* GR-1 and *L. reuteri* RC-14 [50,69,70], with the aim of reducing abnormal microbiota and recurrent dysbiosis.

Other probiotic genera administered orally, such as *Bifidobacterium* spp., can be used in fertility disorders. Zhang et al. [20] managed to modulate the levels of sex hormones in patients with PCOS through the intestine–brain axis with the probiotic strain *Bifidobacterium lactis* V9. In this review, only one article using *Bifidobacterium* in combination with *Lactobacillus* strains showed a specific impact on embryo transfer success; however the authors claimed that the supplementation of probiotics after oocyte retrieval did not improve vaginal colonization or pregnancy rate [52]. The use of the specific probiotic strains for such dysbiosis was corroborated through modifying bacterial vaginosis parameters [46,48,49,50]. There was a probiotic strain in combination with oral metronidazole that was not able to reduce BV recurrence [51]. The lack of success in clinical trials with probiotics as modulators of fertility disorders may be due to the efficacy of the probiotic being strain- and disease-dependent, as well as highly reliant on the dose, duration, administration method, and host state [71,72].

In agreement with the qualitative outcomes retrieved, we found a wide variation in the administration pattern of the probiotics used in the investigations selected. The doses administered in the studies collected for the systematic review ranged from ≥10^7^ CFU/day [46] to 2.5 × 10^10^ CFU/day [48]. We found large variations in the administration time between the administration of the probiotic for 1 day [52] and its administration for 12 weeks [51]. Except for these two studies [51,52], the mean administration time found in the review was 1 week. When we compare these data with the oral administration of probiotics in reproductive disorders [45], it is observed that the range of CFU/day administered is similar to that of vaginal probiotics, from 1 × 10^6^ CFU/day to 3 × 10^10^ CFU/day. However, in terms of treatment duration, the differential range is much more pronounced, varying from 3 to 24 weeks; this could be due to oral probiotics needing a longer duration to reach the natural reproductive site of colonization compared to the more localized site-direct administration for vaginal probiotics. As expected, there were more clinical trials in which probiotics were administered orally (10) than vaginally (5), which fulfilled the high-quality standards for fertility disorders. Until now, bacterial vaginosis has been the most common vaginal syndrome treated by local probiotics, but we consider that well-designed clinical studies would better support and explore the use of vaginal probiotics as therapeutic complementary solutions on reproductive site dysbiosis in relation to unexplained infertility cases.

The meta-analysis outcomes corroborated a slightly modulated vaginal probiotic capacity on the relative abundance of abnormal microbiota. This seemed to be associated with a tendency for microbiome restauration by the level of *Lactobacillus* species. Heterogeneity data were also similar for abnormal microbiota reduction (77%) and restoration of *Lactobacillus* (78%). The data analyzed were in agreement with postulates on the presence of abnormal vaginal microbiota as a factor of recurrent dysbiosis. Vaginal microbiotas of patients with BV contain more diverse and higher counts of *Gardnerella*, *Prevotella*, *Atopobium*, *Mobiluncus*, *Peptostreptococcus*, *Sneathia*, *Leptotrichia*, and *Mycoplasma*, whereas *Lactobacillus* are found in lower quantity and less frequently [58]. The combination of these microbial modifications can synthetize amino compounds and rise the vaginal pH, thus generating a site colonization more prone to several pathogenic infections and vulnerable to unhealthy disorders, including reproductive results [73]. Remarkably, reproductive site *Lactobacillus* species promotion, together with a proportionally decreasing abnormal microbiota, was also supported by in vitro studies that showed lactobacilli inhibiting the colonization of *Gardnerella vaginalis* to the vaginal epithelium tissues and producing bacteriocins, lactic acid, and/or H_2_O_2_, which inhibit the bacteria that cause BV [46].

The limitations of this review are based on the few comparative and qualitative clinical data available because of the low number of eligible studies and limited sample size population. Furthermore, there is no standardized probiotic administration, and there are different doses and several probiotic strains. International guidelines or protocols on probiotics for reproductive-related disease prevention and treatments are required. This will allow for a more significant and unified clinical effect comparison and provide robust meta-analysis outcomes.

In future studies, all probiotics used must be beneficial, safe and harmless to the target patients [74]. In this sense, next-generation probiotics (NGP) have been extensively characterized regarding their physiological interaction with the host [75,76]; therefore, new clinical studies with NGP might better modulate reproductive dysbiosis. Another innovative therapeutic method for reproductive site microbiota modulation could be vaginal microbiota transplantation (VMT) [77,78,79]. This method opens the door to a BV treatment that requires more research to advance it from conceptual analysis to clinical application.

## 5. Conclusions

The present study revealed that only a few clinical trials administering vaginal probiotics for fertility-related dysbiosis applied harmonized protocols for the most common reproductive disorder, bacterial vaginosis. *Lactobacillus acidophilus* remains the first election probiotic species to be vaginally administered. The impact of quantitative microbiota modulation capacities in reproductive-health-related dysbiosis was similar within the selected studies, as proved by the meta-analysis outcomes in which the administration of vaginal probiotics moderately modulated the relative abundance of abnormal microbiota, coinciding with an increase in *Lactobacillus* species. The variety of fertility disorders treated with vaginal probiotics found was significantly low compared to oral administration of probiotics. Hence, future vaginal intervention studies with next-generation probiotics could redirect the effort to obtain not only modulation of microbial biomarkers, but also better holistic reproductive health effects.

## Figures and Tables

**Table 1:** Population, intervention, comparison, and outcome (PICO) criteria for inclusion of studies.

Parameters	Inclusion Criteria
Population	Human
Intervention	Probiotics strains and doses
Comparison	Vaginal probiotics versus placebo
Outcome	Fertility parameters
Setting	Clinical trials (CTs)

**Table 2:** Effects of vaginal probiotic strains administered in clinical trials in reproductive and fertility-related disorders and their relevant clinical results.

Reference	Population Sample (*n*)	Probiotic Strains	Probiotic Doses	Probiotic Administration Time (Weeks)	Disorders/Diseases	Clinical Effects and Health Parameter Modifications
Oerlemans et al. [48]	20 women with vulvovaginal candidiasis (VVC)	*Lactobacillus pentosus* KCA1, *Lactobacillus plantarum* WCFS1, and *Lactobacillus rhamnosus* GG	2.5 × 10^9^–2.5 × 10^10^ CFU/day	1.5	Vulvovaginal candidiasis	Probiotic formulation restores the vaginal microbiota in 45% of women. The other 55% of women needed rescue medication (fluconazole), but, at the end of the study, these women presented a larger reduction in the amount of *Lactobacillus* sp. compared to the other group.
Mastromarino et al. [49]	39 women	Florisia^®^: *Lactobacillus brevis* (CD2), *Lactobacillus salivarius* subsp. *salicinius* (FV2), and *L. plantarum* (FV9)	≥10^9^ CFU/day	1	Bacterial vaginosis	This probiotic product of exogenous strains of *Lactobacillus* spp. administered intravaginally restored the healthy vaginal microbiota and it can be administered to treat bacterial vaginosis (BV) disorders.
Bisanz et al. [50]	14 postmenopausal women	*L. rhamnosus* GR-1 and *Lactobacillus reuteri* RC-14	2.5 × 10^9^ CFU/day	3 (day)	Bacterial vaginosis	Total *Lactobacillus* increased and the proportion of *Atopobium* decreased. In addition, there was a trend for *Gardnerella* and *Prevotella* reduction. No changes in Nugent score and host metabolome.
Bradshaw et al. [51]	450 healthy women	*Lactobacillus acidophilus* KS400	>10^7^ CFU/day	12	Recurrent bacterial vaginosis	*Lactobacillus acidophilus* KS400 administered vaginally in combination of oral metronidazole during an extended course did not cure recurrent bacterial vaginosis.
Gilboa et al. [52]	117 women	Probiotic Femina^®^: *Lactobacillus acidophilus*, *Bifidobacterium bifidum*, and *Bifidobacterium longum*	6 × 10^9^ CFU/treatment	1 (day)	In vitro fertilization (IVF)–embryo transfer cycle	Probiotic Femina^®^ did not affect the vaginal colonization of *Lactobacillus* during oocyte retrieval or embryo transfer and did not improve the pregnancy rate.
